# The Role of Wettability on the Response of a Quartz Crystal Microbalance Loaded with a Sessile Droplet

**DOI:** 10.1038/s41598-019-53233-y

**Published:** 2019-11-21

**Authors:** Brandon Murray, Shankar Narayanan

**Affiliations:** Rensselaer Polytechnic Institute, Department of Mechanical Aerospace and Nuclear Engineering, 110 8th Street, Troy, NY 12180 USA

**Keywords:** Chemical engineering, Mechanical engineering

## Abstract

In this work, the interaction between a sessile droplet’s contact angle and a quartz crystal microbalance (QCM) is elucidated. We differentiate the QCM’s frequency response to changes in the droplet contact area from variations in the dynamic contact angle. This is done by developing a computational model that couples the electrical and mechanical analysis of the quartz substrate with the visco-acoustic behavior of the sessile droplet. From our analysis, we conclude that changes in the contact angle have an effect on the frequency response of the QCM when the droplet height is on the order of the viscous decay length or smaller. On the other hand, changes in the interfacial contact area of the sessile droplets have a significant impact on the frequency response of the QCM regardless of the droplet size.

## Introduction

**Figure 1:**
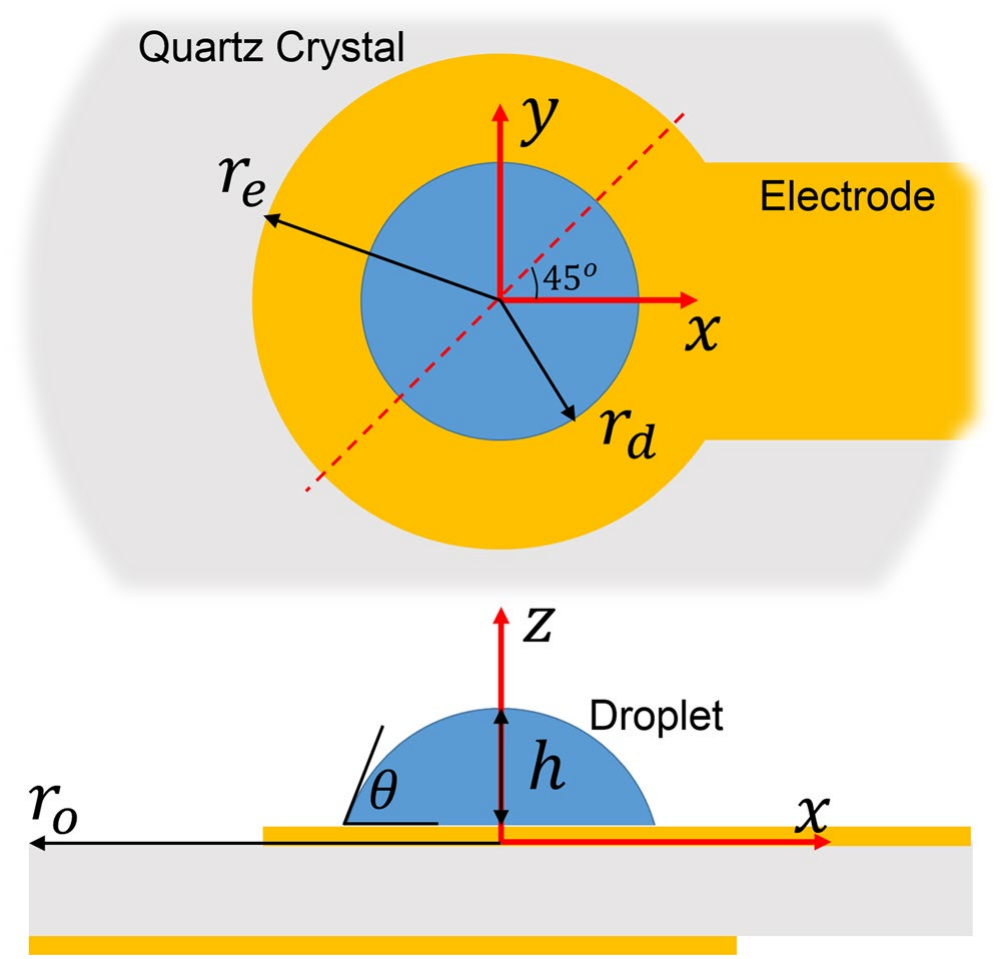
Cross-section and top views of QCM with a centrally placed sessile droplet. The computational study of QCM response considers an AT-cut quartz crystal, coated with concentric electrodes on both sides.

Droplets play an important role in numerous applications such as fuel combustion, spray painting, evaporative cooling, droplet microfluidics, and manufacturing. They are also important in naturally occurring phenomena such as precipitation and dew formation. Consequently, understanding the interaction of droplets with various surfaces, especially their dynamic wetting behavior, is essential. One of the techniques to study the droplet wetting characteristics involves the use of a quartz crystal microbalance (QCM). QCMs are fabricated using quartz crystals that are cut along a specific crystallographic plane, and patterned with metallic electrodes (e.g., Fig. 1). When excited with an oscillating voltage, an unloaded crystal can be made to resonate at its fundamental frequency, *f*_0_. While an unloaded QCM is stress-free, it experiences damping when submerged in a liquid or loaded with a droplet. The viscoelastic coupling between the liquid and the crystal decreases the resonant frequency (*f*) of the QCM. Taking advantage of the QCM’s sensitivity and its frequency response to surface phenomena, wetting and transport characteristics have been studied, which includes droplet spreading^1–3^, contact angle^4^, surface tension^4,5^, and electrowetting^5,6^. Additionally, QCMs have also been used to determine viscosity^7–9^, evaporation^10–13^ and condensation^14^ kinetics and for quantifying slip phenomenon^15–20^.

Although droplets have been studied extensively, it is not clear how surface wettability affects the response of a QCM. In particular, a quantitative comparison of the roles of the droplet contact angle and contact area on the QCM response is lacking. For example, Joyce *et al*. studied the evaporation of alcohols and saw unique frequency signatures corresponding to each liquid^13^. In this study, the authors describe the frequency response of the QCM during constant contact angle and constant area stages. These stages of evaporation were also noted by Picknett and Bexon^21^ for small droplets evaporating in quiescent, low vapor pressure environments, and by Bourgès-Monnier and Shanahan^22^ who showed that these stages depend on the surface and the ambient conditions. However, in the work by Joyce *et al*., the role of wettability was not addressed explicitly since no quantitative relationship between the frequency response and the contact angle was given for droplets evaporating in a constant area mode^13^. Lin and Ward showed that the frequency response to a liquid partially covering the QCM surface is related to the contact area^4^. However, contact angles could only be calculated assuming droplet to be a spherical cap if the volume of the drop was also known. Any dependence on the frequency response from a change in contact angle alone was not addressed. Pham and coauthors used a QCM to study the interfacial effects of drying colloidal suspensions and noted that the response from the constant contact area evaporation stage is essentially constant, indicating that the QCM response could be independent of the contact angle^12^.

Contrary to the studies indicating that the QCM response is only a function of the droplet contact area, Zhuang *et al*.’s characterization of dynamic wetting attributed a change in the contact angle to the frequency response of the QCM^3^. Similarly, Bai and Hang concluded that the mismatch in their experimental and theoretical frequency shifts was likely due to the variation in the contact angle^23^. Prior studies have also been noncommittal on the role of wetting characteristics. For example, when Courtier *et al*. investigated the stages of evaporation using the QCM, they noted a weak increase in the frequency response when the droplet radius was seemingly pinned^11^. Since this was generally attributed to the effects at the edge of the droplet, it was not clear if the change in the frequency response was due to changing contact angle or microscopic variations in the contact area of the droplet. Similarly, while Shin *et al*. were able to correlate the frequency response to the mass of a droplet, for a single particular fluid, they described the need for further studies to understand the effect of contact angle on the QCM response^24^ as they did not consider the viscosity, density or area coverage of their droplets.

In summary, although the QCM has attracted significant interest in studying droplets, it is not clear how wetting characteristics affect the frequency response. Understanding this aspect is crucial for using QCM to quantify mechanisms such as evaporation, condensation, and dynamic wetting behavior. One of the main reasons for the lack in understanding is the challenge associated with conducting an experiment that can successfully decouple the effects of contact angle and contact area of a droplet. In this work, we decouple these effects with detailed computational modeling combined with experiments to capture the QCM response. One of the crucial outcomes of this study is identifying the range of contact angles that can be sensed by a QCM. We found this to depend on the QCM characteristics, the liquid properties, and the size of the droplet.

## Results

### Effect of droplet-surface interaction on the QCM response

**Figure 2:**
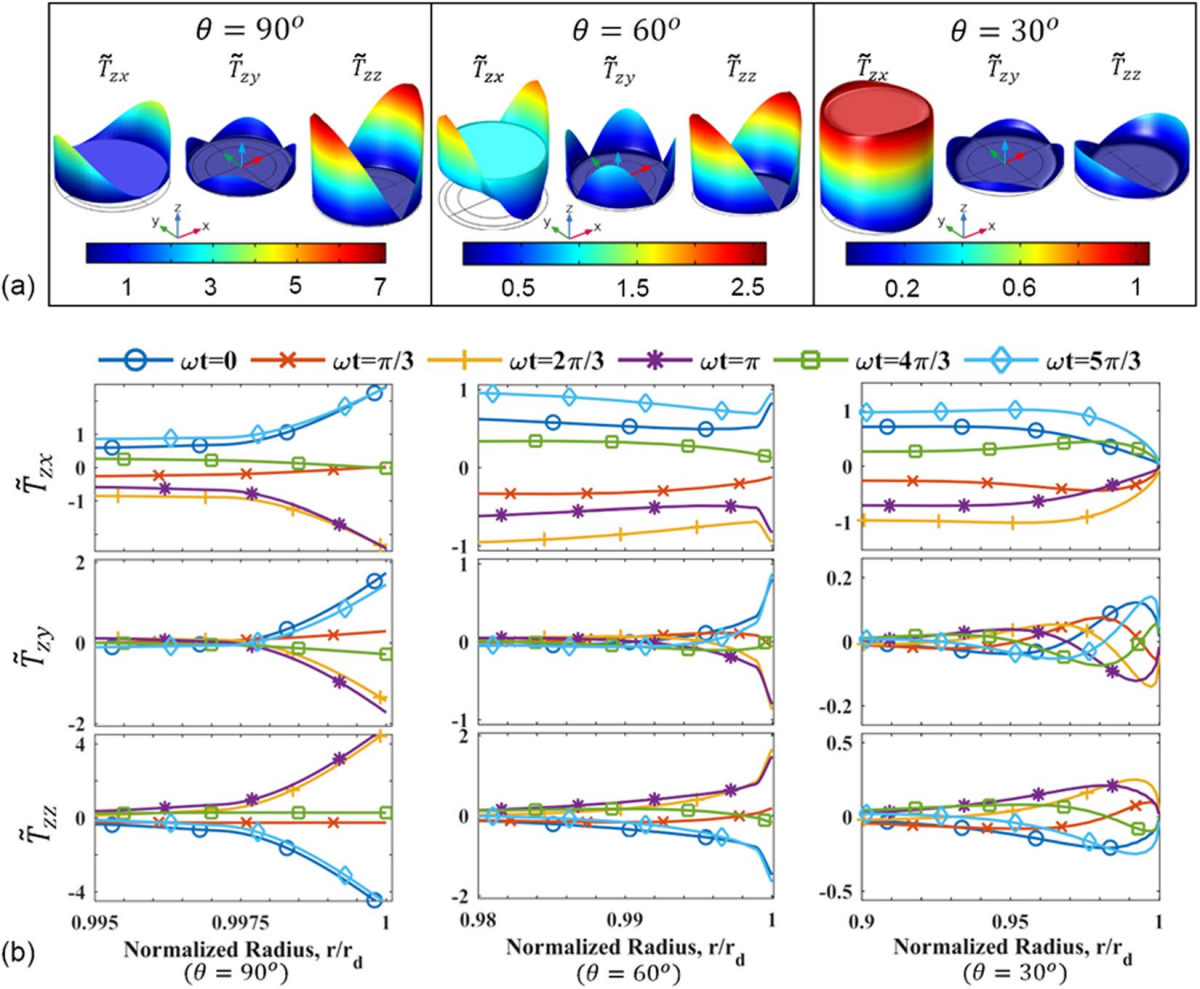
(**a**) Contour plots of normalized ${\tilde{T}}_{zy}$, and ${\tilde{T}}_{zz}$ for contact angles of 90°, 60°, and 30°. (**b**) Normalized ${\tilde{T}}_{zx}$, ${\tilde{T}}_{zy}$, and ${\tilde{T}}_{zz}$ as a function of radial location at different phases of oscillation for different contact angles. The normalized stresses are considered along an axis at 45° from the x-axis on the x-y plane.

Using computational modeling, the frequency response, Δ*f* = *f* − *f*_0_, of the QCM was obtained for a water droplet of radius 10 *μ*m and moderate contact angles of 30°, 60°, and 90°. The shift in resonant frequency from the natural frequency of the crystal $({f}_{0}\approx 10\,{\rm{MHz}})$ is shown in Table 1. In this case, since the frequency shifts are almost identical, the model indicates a negligible effect of the contact angle on the QCM response. This can be explained based on the droplet-QCM interaction, as shown in Fig. 2, which compares the computed stresses at the droplet-QCM interface at different contact angles and phases (*ωt*) of oscillation. The stress components are normalized by the magnitude of shear stress exerted by an equivalent semi-infinite liquid on the QCM (Eq. (14)). Figure 2(a) compares the normalized stress components, ${\tilde{T}}_{zx}$, ${\tilde{T}}_{zy}$ and ${\tilde{T}}_{zz}$, and Fig. 2(b) shows the normalized stress components along an axis oriented at 45° from the *x*-axis on the *xy*-plane (see Fig. 1). For the crystal oscillating along the *x*-direction, the dominant stress is ${\tilde{T}}_{zx}$, which is non-zero across the entire droplet. For a significant portion of the droplet area, ${\tilde{T}}_{zx}$ is identical for all the contact angles. On the other hand, ${\tilde{T}}_{zy}$ and ${\tilde{T}}_{zz}$ are relatively small compared to ${\tilde{T}}_{zx}$ for a large portion of the droplet at all phases of oscillation. However, in the contact line region, ${\tilde{T}}_{zy}$ and ${\tilde{T}}_{zz}$ are also prominent, and all the components of stress show contact angle dependence (animations of the oscillating stress components are provided in the Supplementary Information).

**Table 1:** Frequency response of a water droplet of radius 10 cm and contact angles of 30°, 60° and 90° calculated from the computational model (f_0_ ≈ 10 MHz).

Contact Angle	*θ *= 30°	*θ* = 60°	*θ* = 90°
Frequency Response (Hz)	−4.9689 × 10^−2^	−4.9682 × 10^−2^	−4.9683 × 10^−2^

**Figure 3:**
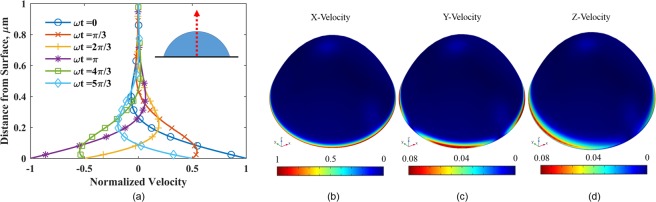
(**a**) Normalized x-velocity along the vertical z-axis of a 60° droplet of 10 *μ*m radius. Computational results are shown with solid lines and theoretical values (Eq. (12)) with dashed line and symbols. The maximum percent difference between any two corresponding curves is 0.08%. x- (**b**), y- (**c**) and z-velocity (**d**) components for a 60°, 10 *μ*m droplet, normalized to maximum velocity.

Complementary to the stress profiles, the velocity in the droplet is dominated by the shear wave along the x-direction, which arises from the motion of the QCM surface. As the crystal oscillates, it drags the liquid close to the surface in phase with it. Away from the surface, the velocity in the liquid decays and is out of phase with the oscillating crystal. Figure 3 shows the computed x-velocity along the axis of the droplet (*z*-axis) plotted at various phases (*ωt*) along with the theoretical velocity described by Stokes’s second problem for a semi-infinite fluid (Eq. (12)). It can be seen that the computed velocity at the center of a small droplet matches the theoretical prediction. The x-velocity decays quickly in the vertical direction and does not change much in the radial and angular directions, as shown in Fig. 3(b). For a chosen liquid, a decay length (δ) can be defined based on the distance from the surface where the shear wave amplitude decays to *e*^−1^ of its value at the crystal surface (Eq. (3)). For water on a 10 MHz crystal, *δ* ≈ 170 nm. This parameter, as shown later, is important in determining if a QCM can sense variations in the contact angle of a droplet.

Compared to the x-velocity, the y and z components are an order of magnitude smaller and prominent only near the contact line where the height of the droplet surface is comparable to the decay length, *δ*. The changes in the velocity components near the contact line explain the deviation of the stress values from that of a semi-infinite liquid for which ${\tilde{T}}_{zx}=1$, ${\tilde{T}}_{zy}=0$ and ${\tilde{T}}_{zz}=0$. These variations in the stress profiles are important to determine the effect of contact angle on the frequency response of the QCM. For a 10 *μ*m water droplet with moderate contact angles on a 10 MHz crystal, although the stress profiles have notable differences, the variation is only near the contact line of the droplet (0.95 < *r*/*r*_d_ < 1). As a result, the difference in the overall shear stress is insufficient to affect the frequency response significantly.

**Figure 4:**
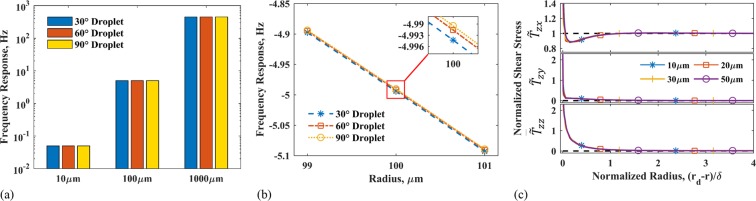
(**a**) Frequency response of different contact angles at various radii. (**b**) Frequency response for different contact angles, and contact radii close to 100 *μ*m. Inset shows the frequency shift for a 100 *μ*m droplet and different contact angles. (**c**) Normalized stress for a droplet of contact angle 90 and different radii. Normalized stress is calculated along a line 45 from the x-axis.

It can be argued that the lack of variation in the frequency response is due to the use of a droplet (*r*_*d*_ = 10 *μ*m) much smaller than the QCM electrode (*r*_*e*_ = 2.5 mm). In particular, droplets with larger contact line region can be expected to show a noticeable difference in the frequency response for different contact angles. In order to probe this aspect, the computational model was used to obtain the frequency response for water droplets of radius 10, 100, and 1000 *μ*m and contact angles of 30°, 60°, and 90° on a 10 MHz quartz crystal. Figure 4(a) shows the computed shift in the resonance frequency (Δ*f*) of the QCM for different contact angles and radii.

Evidently, large changes in the droplet radius (10 *μ*m → 100 *μ*m → 1000 *μ*m) cause distinct frequency variations. However, for a given radius, a change in the droplet contact angle alone results in much smaller variation in the frequency response of the QCM. By investigating droplets around 100 *μ*m, a comparison of the frequency response from a changing radius versus a changing contact angle can be seen in Fig. 4(b). Any variation in the frequency response from a change in the contact angle is dwarfed in comparison to a 1 *μ*m change in droplet radius. For large droplets, where the height, as well as the radius, are much larger than the decay length (*δ*), the frequency responses for a fixed radius and different contact angles are almost identical. Hence, large droplets do not seem to amplify the effect of contact angle on the frequency response. In order to understand why this takes place, the stress exerted at the contact line region is compared to the overall stress at the droplet-QCM interface.

Figure 4(c) shows the normalized stress components (${\tilde{T}}_{ij}$) at the droplet-QCM interface (Eq. (19a)). The normalized stress components are shown in the contact line region for droplets of 90° contact angle and different radii. For large droplets, it is apparent that the shear stress, ${\tilde{T}}_{zx}$, exerted at droplet-surface interface matches that of a semi-infinite liquid as we move away from the contact line region and closer to the droplet center. In the contact line region, the stress values can differ from the center of the droplet and show contact angle dependence, as seen earlier in Fig. 2. Although the contact line region expands with the size of the droplet, the relative contribution of the stress exerted near the contact line becomes less prominent compared to the overall stress exerted by the droplet on the QCM. In addition, the QCM sensitivity can also deteriorate away from the center of the electrode^1,25,26^ (Eq. (5)). As a result, the QCM is less sensitive towards the edge of large droplets. Therefore, for sufficiently large droplets, a change in the contact angle is imperceptible, and the QCM response can be considered to be a function of the contact radius alone. Droplets with dimensions (height, radius) on the order of the decay length (*δ*) may show a frequency response to a changing contact angle with a constant radius.

### Regime map for contact angle-dependent frequency response

**Figure 5:**
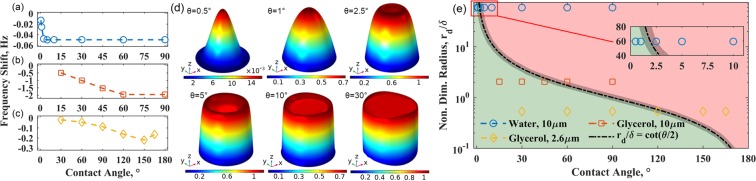
Frequency response of (**a**) 10 *μ*m radius water, (**b**) 10 *μ*m radius glycerol, and (**c**) 2.6 *μ*m radius glycerol droplets. (**d**) Normalized ${\tilde{T}}_{zx}$ stress profiles on the surface of a 10 *μ*m radius water droplet at various contact angles. (**e**) Regime map of contact angle dependent frequency response. The line ${r}_{d}/\delta =\,\cot (\theta /2)$ separates droplets with frequency responses that show contact angle dependence from those that show responses independent of contact angle variations.

For the water droplets considered, while the analysis has not indicated any dependence of the frequency response on the contact angle, this observation cannot be generalized. Indeed, it is possible to detect variations in the contact angle using a QCM for specific combinations of liquids, droplet sizes, and QCM characteristics. To determine conditions suitable for sensing contact angles with a 10 MHz crystal, water (*δ* ≈ 170 nm) droplets of 10 *μ*m radius with contact angles of 0.5° to 90°, and glycerol (*δ* ≈ 5 *μ*m) droplets of 2.6 *μ*m and 10 *μ*m radii with contact angles of 15° to 165° were analyzed. Figure 5(a–c) shows the frequency responses for the various droplets.

At a fixed radius, a shift towards lower resonance frequency (Δ*f* = *f* − *f*_0_) can be observed as the contact angle increases, which can gradually taper off, making higher contact angles indistinguishable. For a 10 *μ*m water droplet, the response stops changing beyond 2.5° (Fig. 5(a)). For a 10 *μ*m glycerol droplet, the response is invariant beyond 60° (Fig. 5(b)). And for a smaller glycerol droplet (*r*_*d*_ = 2.6 *μ*m), the resonance frequency decreases until *θ* ≈ 150° and then increases again, giving the same response at 165° as 120° (Fig. 5(c)).

For droplets with dimensions approaching the viscous decay length (*δ*), as the contact angle changes, the stress profiles vary and differ significantly from the bulk value corresponding to a semi-infinite liquid. For a water droplet (*r*_*d*_ = 10 *μ*m), Fig. 5(d) illustrates this aspect when the contact angle is varied between 0.5° to 2.5°. With characteristic dimensions (*h*, *r*_*d*_) larger than the viscous decay length, the stress profiles for large droplets approach the bulk value at the center of the droplet, as shown in Fig. 5(d), for contact angles varied from 5° to 30°. Consequently, the frequency response becomes invariant to the changes in the contact angle. By extension, it is easy to see how the QCM response to other liquids, like glycerol, may show higher sensitivity to changes in contact angle. With a decay length 30 times larger than that of water, contact angles as large as 60° can be sensed for a 10 *μ*m glycerol droplet.

This understanding can be used to derive a general criterion for the measurable range of contact angles based on a comparison of the droplet size and the viscous decay length. Geometrically, the radius (*r*_*d*_) and the height (*h*) of a droplet are related to the contact angle as ${r}_{d}=h\,\cot (\theta /2)$. We note that the variations in the droplet contact angle can be sensed by the QCM when the droplet height is less than the decay length. Mathematically, the measurable range of contact angles is then given by $0 < \theta  < 2{\cot }^{-1}({r}_{d}/\delta )$. This criterion is shown as a dashed curve in Fig. 5(e) denoting ${r}_{d}/\delta =\,\cot (\theta /2)$ to highlight the region where contact angle effects can be sensed using the QCM. To test this criterion, the frequency responses corresponding to water and glycerol droplets shown in Fig. 5(a–c) are plotted in Fig. 5(e). The data points in Fig. 5(a–c) showing contact angle dependence satisfy this criterion and lie on the left side of the dashed curve, whereas data points that do not show contact angle dependence lie on right side of the dashed curve in Fig. 5(e).

Clearly, *r*_*d*_/*δ* controls the measurable range of contact angle. As *r*_*d*_/*δ* grows with larger droplets, the maximum contact angle that can be detected by the QCM decreases. In this regard, a larger decay length (*δ*) would allow studying contact angle effects for larger droplets. Apart from the thermophysical properties of the liquid, since *δ* is related to QCM characteristics (Eq. (3)), crystals with lower natural frequencies may allow studying the contact angle effects of larger droplets. Finally, it is important to note that Fig. 5(e) is not intended to be a precise cut-off. However, it provides a useful guideline to determine if the frequency response of the QCM is a function of contact angle (Δ*f* = *f*(*θ*, *r*_*d*_)) or mainly a function of contact radius (Δ*f* ≈ *f*(*r*_*d*_)).

### Model prediction versus experiments

**Figure 6:**
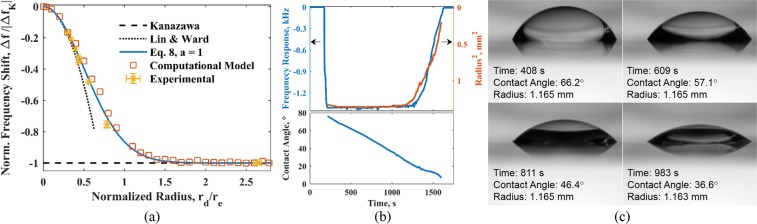
(**a**) Effect of droplet radius on frequency response, normalized to electrode radius and the frequency response a QCM with a semi-infinite liquid (|Δf_K_|l). Error bars represent one standard deviation and are smaller than the marker for the first six experimental data points. (**b**) The frequency response of a 2.5 *μ*l evaporating sessile droplet compared with radius and contact angle measurements. (**c**) Visualization of an evaporating sessile droplet on 10 MHz QCM.

For a crystal (*f*_0_ = 10 MHz) loaded with large water droplets (*r*_*d*_ > 1 mm), since moderate contact angles have a negligible effect on the frequency response (Fig. 5(e)), the response can be approximated as a function of droplet radius (Eq. (8)). Figure 6(a) shows the shift in the resonance frequency of the QCM for droplets of different radii using Eq. (8). Also plotted are the frequency responses obtained from the computational model and experiments, showing good agreement with Eq. (8). In this case, experimental results were obtained by recording the radius of a water droplet on a horizontal 10 MHz QCM with gold electrodes using a commercial system (eQCM, Gamry Instruments) and overhead imaging using a Nikon D610 DSLR and InfiniProbe TS-160 macro lens. Deionized water droplets (Sigma Aldrich) of known volume were deposited at the center of the electrode multiple times for repeatability. The frequency response for small droplets was also predicted using Lin and Ward’s study^4^, which uses a first-order approximation, and Kanazawa’s equation (Eq. (2)). Since the effect of contact angle can be neglected for the droplet dimensions considered, the computational predictions and experimental results agree well with Eq. (8). For small droplets $({r}_{d}\ll {r}_{e})$, there is a good agreement with Lin and Ward’s study. As droplets become larger, the response approaches Kanazawa’s equation, which is applicable for semi-infinite liquids.

For large droplets, the contact angle independence and radius dependence was also observed in experiments involving evaporation of water droplets on the QCM. In this case, the frequency response of the evaporating sessile droplet was determined using a commercial system (eQCM, Gamry Instruments) with a 10 MHz AT-cut crystal. Figure 6(b) shows the characteristic curve of frequency response over time. These experiments were conducted while observing the change in the shape of the droplet, as shown in Fig. 6(c). Visualization allowed monitoring the radius of the droplet versus time^27^, which is plotted in Fig. 6(b) along with the frequency response. Note that similar curves were also reported in earlier studies without a definitive conclusion regarding the dependence of QCM response on the contact angle^11–13,28^.

The visualization clearly shows a constant contact radius mode of evaporation. From the deposition of the droplet until *t* = 1200 seconds, the radius measured and shown in Fig. 6(b), is constant during this period while the contact angle changes from almost 80° to 30°. As anticipated from the regime map (Fig. 5(e)) and the computational results shown in Fig. 4(a,b), the large variation in the contact angle of a 1 mm sized droplet produces no discernible frequency response of the QCM. As the contact line de-pins and the radius begins to decrease, the frequency increases proportionally. This observation is in agreement with the computational model, which confirms that the QCM is ineffective in keeping track of any change in the contact angle for larger droplets, but is sensitive to changes in the contact area of a droplet.

## Conclusions

Although QCMs have been used widely to study droplets on different surfaces, it was not clear how the contact angle or wettability of a droplet affects the frequency response of the QCM. In this study, the roles of droplet contact angle and contact area on the frequency response of a QCM were determined using detailed computational modeling supported with experiments. The computational model solves the piezoelectric (quartz) and fluid (droplet) domains, which allows predicting the frequency response of droplets with different contact angles and radii. The model indicates a negligible change in the resonance frequency of the QCM when the contact angle of a 10 *μ*m droplet was varied from 30° to 90° on a 10 MHz AT-cut quartz crystal. To see if larger droplets show similar behavior, the model predicted the frequency response of water droplets with a radius of 10, 100, and 1000 *μ*m and contact angles of 30°, 60°, and 90°. For these droplets, the stress profiles at the droplet-QCM interface show notable differences when the contact angle is changed. However, the variations in the stress profiles are confined to the contact line region of the droplet, and less prominent compared to the overall stress exerted at the droplet-QCM interface. Hence, the QCM would not sense any change in the contact angle. However, it is quite sensitive to changes in the droplet radius.

The conditions suitable for sensing variations in the droplet contact angles were found to depend on the viscous decay length, *δ*. By comparing the droplet geometry with *δ*, it is possible to estimate the range of contact angles that can be sensed by the QCM. This study found the measurable range of contact angles to be $0 < \theta  < 2{\cot }^{-1}({r}_{d}/\delta )$. Consequently, for large droplets, the QCM response was found to depend mainly on the droplet radius. Using visualization of evaporating droplets, we found similar behavior in experiments, wherein no change in frequency was observed when the droplet was pinned, and the contact angle was decreasing. However, the resonance frequency increased significantly as the droplet receded during evaporation. In summary, this study provides a more in-depth insight into the role of wettability on the frequency response of a QCM, which will allow interpreting data from a QCM accurately to elucidate mechanisms like evaporation, condensation, and droplet spreading.

## Background Theory

The change in the resonance frequency, Δ*f*, of the QCM due to the deposition of a thin, rigid mass, Δ*m*, on the crystal is given by Sauerbrey^29^ (Eq. (1)), where ${\rho }_{q}$ is the density and ${\mu }_{q}$ is the shear modulus of quartz, and *A*_*q*_ denotes the active or electrode area of the quartz crystal.

1 $$\Delta f=-\frac{2{f}_{0}^{2}}{{({\rho }_{q}{\mu }_{q})}^{1/2}}\frac{\Delta m}{{A}_{q}}=-\,{C}_{f}\frac{\Delta m}{{A}_{q}}$$

**Figure 7:**
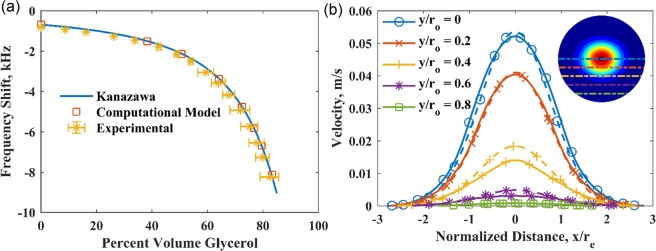
(**a**) Comparison of computational frequency response to Kanazawa’s equation and experimental results. (**b**) Comparison of computed velocity (solid) of a wetted QCM along lines parallel to the x-axis to a fitting of Eq. 5 with the parameters *v*_*c*_ = 0.0536 m/s and *a* = 0.8523 (dashed). Inset showing contour plot of velocity across the QCM surface indicates weak angular dependence of velocity. Dotted lines on the contour plot correspond to y-locations plotted in the main figure.

An unloaded QCM can be considered to be stress-free. When submerged in a semi-infinite liquid of density, ${\rho }_{l}$, and viscosity, *μ*_*l*_, the viscoelastic coupling between the liquid and the QCM will decrease the resonant frequency of the crystal. This decrease is derived from a force-balance by Kanazawa^30,31^ (Eq. (2)), which is seen experimentally and predicted computationally, as shown in Fig. 7(a).

2 $$\Delta f=-\,\frac{{f}_{0}^{3/2}}{{(\pi {\rho }_{q}{\mu }_{q})}^{1/2}}{({\rho }_{l}{\mu }_{l})}^{1/2}$$

Note that the total mass of liquid on the crystal does not play a role in Eq. (2). As the crystal oscillates, it drags the liquid close to the surface in phase with it. Away from the surface, the velocity in the liquid decays and is not in phase with the oscillating crystal. Mathematically, this is similar to the Stokes problem, which describes the velocity in the liquid as a damped sinusoid. For a QCM oscillating at an angular frequency, *ω*, the effective decay length, *δ*, is given by the following equation^4^. For a 10 MHz crystal submerged in water, *δ* is 170 nm.

3 $$\delta ={(\frac{2{\mu }_{l}}{\omega {\rho }_{l}})}^{1/2}$$

By comparing Eqs. (1) and (2), an effective mass layer for a semi-infinite liquid can be determined. Considering the areal mass density, Δ*m*/*A*_*q*_, to be $\delta {\rho }_{l}/2$, Eqs. (1) and (2) can be found to be analogous^4^. Hence, the effective mass layer and the sensing capability of a QCM in a liquid media extends only to a distance of about *δ*/2 from the surface of the QCM. Consequently, any phenomena occurring well beyond *δ*/2 in the liquid are typically not sensed. However, there are exceptions to this rule. Factors known to affect the frequency response of the QCM include surface roughness^12,32^, hydrostatic pressure^33^, and the conductivity of the liquid^34,35^. Under certain conditions, each of these effects can be minor. For example, with smooth polished surfaces and small droplets, roughness and gravitational effects can be neglected. In the case of liquids, like deionized water, the electrical conductivity is typically low (~5.5 × 10^−6^ S/m), which is too small to influence the frequency response of the QCM^35^.

Additionally, for liquids, longitudinal or compressional waves are also found to exist due to the nonuniform velocity profile at the surface of the QCM^36–38^. For a finite fluid layer with a liquid-air interface, this can impact the frequency response due to the formation of stationary or standing waves between the QCM surface and the liquid-air interface^39^. While it is possible to see the effect of compressional waves on the frequency response, it only occurs at specific radii and heights. The response is magnified when the droplets are placed off-center relative to the center of the QCM electrode^28,39^. Since the effect of compressional waves is minimal at the center of the electrode^36,37^, they are insignificant for small droplets (${r}_{d}\ll {r}_{e}$) centered on the QCM. In addition, the compressional waves become less prominent for quartz crystals with higher natural frequencies (*f* > 4 MHz) and those with planar crystal geometries^39^.

Compared to a semi-infinite liquid, the frequency response to a finite liquid volume is different, which can be derived as follows. The point mass sensitivity, *c*_*f*_, of the QCM is known to depend on the surface velocity, *v*_*s*_, as ${c}_{f}(r,\theta )\propto |{v}_{s}(r,\theta ){|}^{2}$, where both *c*_*f*_ and *v*_*s*_ are expressed in cylindrical polar coordinates^25^. The velocity profile of a QCM can be considered to be a Gaussian function centered at the origin (Eq. (4)), as assumed in many studies^1,25,26^.

4a $${v}_{s}(r,\theta ,t)={v}_{c}\,{{\rm{e}}}^{((a-b){\cos }^{2}\theta -a)\frac{{r}^{2}}{{r}_{e}^{2}}}\,\cos (\omega t)$$

4b $${v}_{s}(r,t)\approx {v}_{c}{{\rm{e}}}^{-a{r}^{2}/{r}_{e}^{2}}\,\cos (\omega t)$$

In Eq. (4), *v*_*c*_ is the velocity at the center of the electrode, *a* and *b* are intrinsic QCM constants, *r* is the radial position, *r*_*e*_ is the radius of the electrode, and *ωt* is the phase of the oscillation^40^. For a planar crystal, the angular dependence can be neglected^25^, and with *a* ≈ *b*, the velocity only varies in the radial direction (Eq. (4b)). In this study, we also confirm the existence of a Gaussian velocity profile^1,26^ with computational modeling (Fig. 7(b)). In Eq. (4b), *a* is a constant intrinsic to the QCM. While its value is not precisely determined, some literature report *a* ≈ 2^40,41^, while Lin^1,36^ reports *a* ≈ 1. In this study, *a* is found by a regression analysis of the computed velocity profiles to be near unity, as discussed later.

With the velocity profile known, the point mass sensitivity, *c*_*f*_, can be determined using the area-averaged value of ${v}_{s}^{2}$ (Eq. (4b)) over the QCM surface^25^, as shown in Eq. (5). The Gaussian distribution for QCM sensitivity, as confirmed by Hess^37^, can be slightly ellipsoidal like the surface velocity (Eq. (4a)). However, it can be well approximated by ignoring its angular dependence^25,37^.

5 $${c}_{f}(r)={C}_{f}\frac{2a}{\pi {r}_{e}^{2}}{e}^{-2a{r}^{2}/{r}_{e}^{2}}$$

Further integration of *c*_*f*_ along with the effective mass layer over a portion of the QCM surface will determine the frequency response of the QCM to finite liquid loading^1^,

6 $$\Delta f=-\,\,{\int }_{0}^{2\pi }\,{\int }_{0}^{r}\,{c}_{f}(r^{\prime} ,\theta )m(r^{\prime} ,\theta )r^{\prime} dr^{\prime} d\theta =\pi \delta {\rho }_{l}{\int }_{0}^{r}\,{c}_{f}(r^{\prime} )r^{\prime} dr^{\prime} $$

Here, $m(r^{\prime} ,\theta )$ has been replaced with the effective aerial mass density $(\delta {\rho }_{l}/2)$. By using Eqs. (5) and (6), the frequency response, Δ*f*, for a finite liquid radius can be found as

7 $$\Delta f=-\frac{1}{2}{C}_{f}\delta {\rho }_{l}(1-{e}^{-2a{r}^{2}/{r}_{e}^{2}})$$

In order to write Eq. (7) in a form analogous to Kanazawa’s equation (Eq. (2)), *C*_*f*_ and *δ* can be replaced using Eqs. (1) and (3), respectively.

8 $$\Delta f=-{f}_{0}^{3/2}\frac{{({\rho }_{l}{\mu }_{l})}^{1/2}}{{(\pi {\rho }_{q}{\mu }_{q})}^{1/2}}(1-{e}^{-2a{r}^{2}/{r}_{e}^{2}})$$

Equation (8) describes the frequency shift based on the finite contact radius, *r*, of a fluid on the QCM. For a large contact radius (*r* → ∞), Eq. (8) takes the form of Kanazawa’s equation for a crystal loaded with semi-infinite liquid. However, note that Eq. (8) does not include any information regarding the contact angle of the finite liquid since it is derived assuming a column with a contact radius, *r*, and infinite height. The application of Eq. (8) can be seen in Fig. 6(a). In order to determine the effect of contact angle on the resonant frequency, the force interactions between the crystal surface and the droplet must be examined.

For a droplet on the QCM, the velocity in the fluid domain can be derived from the Navier-Stokes equations, which can be simplified to Eq. (9) under the assumptions that spatial derivatives in the *z*-direction are much larger than spatial derivatives in *x* and *y* directions, and noting that the velocity in the *x*-direction, *v*_*x*_, is much larger than the other velocity components^40^.

9 $${\rho }_{l}\frac{\partial {v}_{x}}{\partial t}={\mu }_{l}\frac{{\partial }^{2}{v}_{x}}{\partial {z}^{2}}$$

With Eqs. (4b) and (10) as boundary conditions and Eq. (11) as the initial condition, the solution of Eq. (9) is nearly identical to Stokes’s second problem, as shown in Eq. (12)^36^. This analytical solution is compared with the computational result in Fig. 3.

10 $${v}_{x}(z\to \infty ,t)=0$$

11 $${v}_{x}(r,t=0)={v}_{c}{e}^{-a{r}^{2}/{r}_{e}^{2}}$$

12 $${v}_{x}(r,z,t)={v}_{c}{e}^{(-a{r}^{2}/{r}_{e}^{2}-z/\delta )}\,\cos (\omega t-z/\delta )$$

For a liquid on the QCM surface, the velocity decays quickly in the *z*-direction. This velocity gradient gives rise to shear stress that damps the motion of the QCM surface. The shear stress, *T*_*zx*_, on the QCM can be calculated from the velocity gradient at the crystal surface as ${T}_{zx}=-\,{\mu }_{l}(\partial {v}_{x}/\partial z){|}_{z=0}$, which gives

13 $${T}_{zx}=|{T}_{zx}|\,\cos (\omega t+\pi /4)$$

where the magnitude of the shear stress, $|{T}_{zx}|$, is given by

14 $$|{T}_{zx}|={v}_{c}{(\omega {\rho }_{l}{\mu }_{l})}^{1/2}{e}^{-a{r}^{2}/{r}_{e}^{2}}$$

Although the stress on the QCM surface is similar in form to the velocity (Eq. (12)), it is out of phase by *π*/4 and is proportional to ${({\rho }_{l}{\mu }_{l})}^{1/2}$. Since the viscous decay length, *δ*, is typically small compared to the height of the droplet, the assumptions in Eq. (9) hold over the vast majority of the contact area between the droplet and the QCM. However, these assumptions do not hold near the contact line where the height of the droplet is comparable to the viscous decay length. In addition, the *y* and *z*-component velocities also become significant near the contact line. Consequently, there is no simple analytical solution for the force or the shear stress on the QCM surface in the contact line region as a function of the contact angle. Hence, a computational model is necessary to derive the force exerted on the QCM surface near the three-phase contact line.

## Computational Modelling of QCM Response

By using computational modeling, the frequency response of the QCM can be determined for droplets of a given radius and contact angle. Specifically, the model predicts the electrical admittance of the QCM over a spectrum of excitation frequencies. The QCM exhibits resonance at the frequency, *f*, where the conductance (the real component of electrical admittance) is maximum.

### Fully-coupled computational model

The fully-coupled model considers a droplet on an AT-cut quartz crystal to investigate the effect of contact angle and contact area on the QCM response (Fig. 1). Dimensions of the 5 MHz and 10 MHz QCMs used in this study are given in the Supplementary Table S3. The three-dimensional transient model directly couples a piezoelectric domain (quartz crystal) to a fluid domain (water droplet). The piezoelectric domain is given a sinusoidal voltage causing it to oscillate in a thickness-shear mode. The oscillations at the solid-liquid interface give rise to highly damped shear and compressional waves in the fluid. In order to characterize the motion of the quartz crystal and the droplet, the computational model solves the governing equations in the piezoelectric and the fluid domains, as described below. To solve this highly coupled and complex system a commercially available finite element software (COMSOL Multiphysics^42^) was used. All governing equations were solved in the frequency domain to capture the oscillatory nature of the QCM and droplet response.

#### Piezoelectric domain

The equation governing the motion of the quartz crystal is given by

15 $${\rho }_{q}\frac{{\partial }^{2}{u}_{q}}{\partial {t}^{2}}+\nabla \cdot T=0$$

where *u*_*q*_ is the displacement vector, ${\rho }_{q}$ is the density of quartz, and *T* is the stress tensor. Since an oscillating potential is applied to the QCM, charge conservation in the quartz crystal requires that $\nabla \cdot D={\rho }_{v}$, where *D* is electric displacement vector and ${\rho }_{v}$ is the volumetric charge density. In order to account for the piezoelectric effect in quartz, a constitutive equation coupling Hooke’s law with Maxwell’s equation is used, as shown in Eq. (16)^43^. This equation relates *T* and *D* with the strain, *S*, and the electric field, *E*.

16 $$[\begin{array}{c}S\\ D\end{array}]=[\begin{array}{cc}{s}^{E} & {d}^{t}\\ d & {\varepsilon }^{T}\end{array}]\,[\begin{array}{c}T\\ E\end{array}]$$

Here, *s*, *d*, and *ε* are material properties denoting the elastic compliance, piezoelectric and permittivity tensors, respectively. The superscripts *E* and *T* denote evaluation at constant electric field and constant stress, respectively, and the superscript *t* denotes transpose^43^. Generally, these properties depend not only on the chosen material but also on the plane along which the crystal is cut. In this study, left-handed quartz property values were obtained from the 1978 IEEE Standard on Piezoelectricity, and rotated at the Euler angles (ZXZ) of *α* = 0, *β* = −54.75°, *γ* = 0 for an AT-cut crystal. These properties are listed in the Supplementary Information^42^. In practice, while the QCM consists of two metallic electrodes, they are neglected in the computational analysis since they are relatively thin and have low resistivity.

#### Fluid domain

The acoustic waves generated at the solid-fluid boundary can be captured by linearizing the continuity, momentum, and energy conservation equations in the fluid domain. For small acoustic perturbations around the steady-state, the equations governing mass and momentum conservation are given by

17 $$\frac{\partial {\rho }_{{l}_{t}}}{\partial t}+\nabla \cdot ({\rho }_{{l}_{0}}v)=0$$

18 $${\rho }_{{l}_{0}}\frac{\partial v}{\partial t}=\nabla \cdot [-\,pI+{\mu }_{l}(\nabla v+{(\nabla v)}^{t})-(\frac{2}{3}{\mu }_{l}-{\mu }_{B})(\nabla \cdot v)I]$$

Assuming an isothermal droplet (*T*_0_ = 25 °C) in atmospheric conditions (*p*_0_ = 101.325 kPa), the dependent variables include the velocity, *v*, and pressure, *p*, which are small periodic perturbations arising from acoustic waves in the fluid. The small acoustic deviations in the fluid density, ${\rho }_{{l}_{t}}$, can be found using the acoustic pressure, *p*, as ${\rho }_{{l}_{t}}={\rho }_{{l}_{0}}{\beta }_{T}p$, where the equilibrium liquid density, ${\rho }_{{l}_{0}}=f({p}_{0},{T}_{0})$ and *β*_*t*_ is isothermal compressibility. Here, *μ*_*l*_ and *μ*_*B*_ denote dynamic and bulk viscosity, respectively. These properties are listed in Supplementary Tables S1 and S2^44,45^.

A fully-coupled dynamic model solves Eqs. (15) to (18). As boundary conditions for the piezoelectric domain, a sinusoidal voltage, $V={V}_{0}\,\cos (\omega t)$ is applied at the bottom or non-sensing electrode of the QCM (Fig. 1), while the top or sensing electrode is grounded (*V* = 0). The remaining areas on the QCM are assigned a zero charge density, or $n\cdot D=0$. At the quartz-droplet interface, a continuity in velocity (no-slip) condition is assigned. The remaining boundaries are left unconstrained. For analyzing droplets, a normal pressure given by the Laplace pressure, 2*σκ*, is applied to the fluid domain at the air-water interface based on the surface tension, *σ*^45^ and the curvature, *κ*. The fully-coupled model is meshed, as shown in Supplementary Fig. S1. Since the fully-coupled model interfaces the piezoelectric and fluid domains directly, analysis of large droplets becomes computationally challenging. For this reason, large droplets are analyzed using a decoupled model where the droplet and the crystal are analyzed separately, and then coupled indirectly, as described below.

### Decoupled computational model

**Figure 8:**
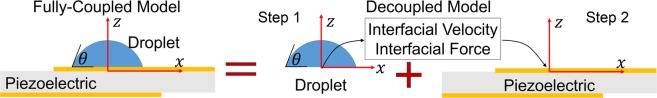
Interfacing the droplet with QCM in a decoupled model.

In the decoupled model, the droplet and the QCM are indirectly coupled with continuity in velocity and stress at the interface, as shown in Fig. 8. In this case, the frequency response to different contact angles and areas can be obtained by mapping the shear stress from an independent droplet analysis to the QCM surface.

The decoupled model obtains a normalized stress profile from a finite element model of a sessile droplet with an oscillating base. The base is prescribed a velocity in the x-direction that is Gaussian in form as experienced by a droplet on a QCM surface (Eq. (4b)). The stress profile (Eq. (19a)) is then determined as a complex-valued function of the polar coordinates and time ($\tilde{r},\theta ,t$). Subscript *ij* in Eq. (19a) denotes *zx*, *zy*, and *zz* directions. A non-dimensional radial coordinate, $\tilde{r}$ (Eq. (19b)), is useful in capturing the stress near the three-phase contact line region for various sized droplets.

19a $${\tilde{T}}_{ij}(\tilde{r},\theta ,t)=\frac{{T}_{ij}(\tilde{r},\theta ,t)}{\sqrt{\omega {\rho }_{l}{\mu }_{l}}|{v}_{s}(\tilde{r},\theta )|}$$

19b $$\tilde{r}=({r}_{d}-r)/\delta $$

The normalized stress components for water droplets of 90° contact angle and different radii are shown in Fig. 4(c). The stresses for all droplets lie on the same curve when non-dimensionalized in magnitude and position by Eqs. (19a) and (19b), respectively. For a given contact angle and viscous decay length, the edge effects due to stress propagate a fixed distance into the droplet from the contact line. This contact line region width is fixed even as the radius of a droplet increases since the geometry (normalized using *δ*) is identical for a given contact angle; thus the normalized fluid velocity and stress profiles will also be similar.

The shear stress profile (${\tilde{T}}_{ij}(\tilde{r},\theta ,t)$) is then mapped on to the QCM surface as a velocity-dependent boundary stress in place of a physical droplet (Eq. (20)). This approach allows efficient calculation of the QCM response by reducing the computational demands significantly.

20 $${T}_{ij}(r,\theta ,t)=-\,{(\omega \rho \mu )}^{1/2}\,{\tilde{T}}_{ij}(\tilde{r},\theta ,t)\,\frac{\partial {u}_{x}}{\partial t}$$

Here, *u*_*x*_ is the displacement of the QCM surface in the *x*-direction, which depends on the potential applied at the QCM electrodes. Analogous to a droplet placed on the QCM, this stress damps the oscillations to result in a shift in the resonance frequency compared to an unperturbed QCM.

#### Model prediction versus experiments and theory

Figure 4(c) shows that the shear stress, *T*_*zx*_, was found to be identical to Eq. (13), derived from theory, for a major portion of the droplet area. The interfacial stress approaches the value of a semi-infinite liquid (Eqs. (13) and (14)) within a distance of approximately *δ* from the contact line. The figure also indicates that all the droplets of the same contact angle exhibit the same non-dimensional form. Hence, Eq. (19a) can be used to extrapolate the shear stress profiles for droplets of any radii sufficiently large $({r}_{d}\gg \delta )$. However, for droplets where the radius or height are comparable to *δ*, the stress cannot be scaled up to other radii because it does not reach the bulk stress value. However, it can still be used for the decoupled analysis of a droplet of similar size. Animations comparing the components of normalized stress calculated using the fully-coupled and the decoupled methods for a 10 *μ*m droplet with 30° and 60° contact angles are provided in the Supplementary Information.

For a semi-infinite liquid loaded on the QCM, the frequency response was predicted using the decoupled model and compared with experiments and theory (Fig. 7). Using *T*_*zx*_ (Eq. (13)) as the boundary condition over the entire QCM surface, the decoupled model predicts the frequency response for liquids with different densities and viscosities. The experiments were performed using QCM200 system (Stanford Research Systems) with a 5 MHz AT-cut quartz crystal of 1-inch diameter, submerged in a temperature-controlled beaker with a water-glycerol mixture (Fisher Chemical). The density and the viscosity of water-glycerol mixture for the experiments and the simulations were obtained from Volk and Kähler^46^ and Cheng^47^. For the range of density and viscosity considered, Kanazawa’s equation (Eq. (2)) was also used to predict the frequency response of the QCM. Figure 7(a) shows good agreement in the frequency shifts predicted using the decoupled model, Kanazawa’s equation, and those observed in the experiments. This shows that the decoupled model can be used to investigate stress-based loads on the surface of a QCM.

The velocity profile obtained from the computational model for a semi-infinite liquid can be compared to the expected profile given by Eq. (4b). For a 10 MHz QCM loaded with water, the value of *a* in Eq. (4b) is 0.852, and *v*_*c*_ = 0.054 m/s. Figure 7(b) shows this nonlinear fitting and the computed velocities along several lines parallel to the x-axis, as indicated by the inset. The computational prediction of QCM’s surface velocity matches well with the expected Gaussian velocity distribution. Note also that the value of *a* obtained in this study is close to the value presumed in the literature (*a* ≈ 1)^1^.

**Figure 9:**
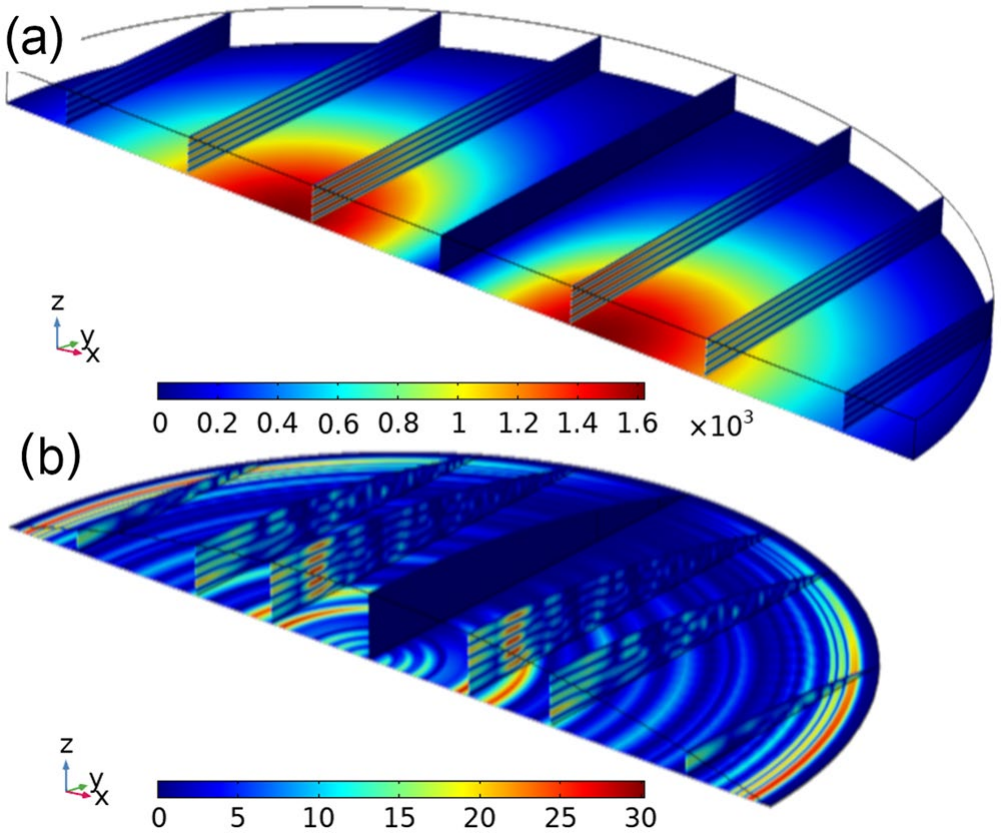
(**a**) Magnitude of acoustic pressure (Pa) from compressional waves in (**a**) a finite liquid layer of height 9*λ*/4 and (**b**) a 5 mm radius droplet where it has a height of 9*λ*/4 over the predicted compressional lobes. Both (**a**,**b**) have symmetry over the x-axis.

Prior research has shown that compressional or standing waves in the liquid can lead to a unique frequency response for droplets of a particular size and placed at specific locations on the QCM^28,39^. For a horizontal water-air interface, resonance based on compressional modes occur at heights of $(2n-1)\lambda /4$, or odd multiples of *λ*/4, which has been shown in the previous efforts^36^. The computational model in this study accurately predicts these resonances. Figure 9(a) shows the pressure waves in a liquid reservoir of height 9*λ*/4 on the QCM surface, surrounded with air and symmetry over the x-axis. In the case of a finite reservoir, the yz-plane has a pressure minimum while there exist two compressional lobes on either side of the center of the electrode along the x-axis. This was also found in prior research^28,36,39^. On the other hand, Fig. 9(b) shows a 5 mm radius droplet with a contact angle of 10.35° that corresponds to a height of 9*λ*/4 over where the compressional modes occur in Fig. 9(a). In this case, the yz-plane still has minimum pressure, but compressional lobes do not appear. There exists only a small region of constructive interference waves. Hence, compressional waves were not found to be a major factor in the droplets, in comparison to the case of a finite liquid reservoir. This was also noted by Couturier^39^, who found high frequency (*f*_0_ > 4MHz) planar crystals to have a weaker frequency response to compressional waves.

## Supplementary information

Supplementary Information

Combined Stress

Nondimensional Wall Stress

Comparing Tzx for Coupled and Decoupled Analysis

Comparing Tzy for Coupled and Decoupled Analysis

Comparing Tzz for Coupled and Decoupled Analysis

## References

[1] Lin Z, Hill RM, Davis HT, Ward MD, Hill RM (1994). Determination of Wetting Velocities of Surfactant Superspreaders with the Quartz Crystal Microbalance. Langmuir.

[2] Lin Z, Stoebe T, Hill RM, Davis HT, Ward MD (1996). Improved Accuracy in Dynamic Quartz Crystal Microbalance Measurements of Surfactant Enhanced Spreading. Langmuir.

[3] Zhuang H, Lu P, Lim SP, Lee HP (2007). Frequency Response of a Quartz Crystal Microbalance Loaded by Liquid Drops. Langmuir.

[4] Lin Z, Ward MD (1996). Determination of Contact Angles and Surface Tensions with the Quartz Crystal Microbalance. Anal. Chem..

[5] Lin, J.-W. & Fan, S.-K. Real time contact angle measurement by quartz crystal microbalance for EWOD studies. In *2010 IEEE 5th International Conference on Nano/Micro Engineered and Molecular Systems*, 325–328 (IEEE, 2010).

[6] Esmeryan KD, McHale G, Trabi CL, Geraldi NR, Newton MI (2013). Manipulated wettability of a superhydrophobic quartz crystal microbalance through electrowetting. J. Phys. D: Appl.Phys..

[7] Saluja Atul, Kalonia Devendra S. (2004). Measurement of fluid viscosity at microliter volumes using quartz impedance analysis. AAPS PharmSciTech.

[8] Ash Dean C, Joyce Malcolm J, Barnes Chris, Booth C Jan, Jefferies Adrian C (2003). Viscosity measurement of industrial oils using the droplet quartz crystal microbalance. Measurement Science and Technology.

[9] Bai, Q., Hu, J., Huang, X. & Huang, H. Using QCM for field measurement of liquid viscosities in a novel mass-sensitivity-base method. In *2016 IEEE International Frequency Control Symposium (IFCS)*, 1–3, 10.1109/FCS.2016.7546819 (IEEE, New Orleans, LA, USA, 2016).

[10] Zhuang H, Lu P, Lim SP, Lee HP (2008). Study of the Evaporation of Colloidal Suspension Droplets with the Quartz Crystal Microbalance. Langmuir.

[11] Couturier G, Vatinel S, Boisgard R, Aimé JP, Chabli A (2009). Quartz crystal microbalance and evaporation of sessile droplets. J. Appl. Phys..

[12] Pham NT, McHale G, Newton MI, Carroll BJ, Rowan SM (2004). Application of the Quartz Crystal Microbalance to the Evaporation of Colloidal Suspension Droplets. Langmuir.

[13] Joyce MJ (2000). Evaporation of sessile drops: Application of the quartz crystal microbalance. Langmuir.

[14] Su J, Charmchi M, Sun H (2016). A Study of Drop-Microstructured Surface Interactions during Dropwise Condensation with Quartz Crystal Microbalance. Sci. Reports.

[15] Kwon S-Y, Kim J-C, Choi B-I (2007). Recognition of supercooled dew in a quartz crystal microbalance dew-point sensor by slip phenomena. Metrol..

[16] Xue Y (2015). How solid-liquid adhesive property regulates liquid slippage on solid surfaces?. Langmuir.

[17] Zhuang H, Lu P, Siak PL, Heow PL (2008). Effects of interface slip and viscoelasticity on the dynamic response of droplet quartz crystal microbalances. Anal. Chem..

[18] Zhuang, H., Lee, H. P. &Lim, S. P. Dynamic response of quartz crystal microbalances in contact with silicone oil droplets. In Quan, C., Qian, K., Asundi, A. K. & Chau, F. S. (eds) *Proceedings of SPIE*, vol. 7522, 1–8, 10.1117/12.848916 (2009).

[19] McHale, G. *et al*. Sensor response of superhydrophobic quartz crystal resonators. In *2008 IEEE International Frequency Control Symposium*, 698–704, 10.1109/FREQ.2008.4623089 (IEEE, 2008).

[20] Roach P, McHale G, Evans CR, Shirtcliffe NJ, Newton MI (2007). Decoupling of the liquid response of a superhydrophobic quartz crystal microbalance. Langmuir.

[21] Picknett R, Bexon R (1977). The evaporation of sessile or pendant drops in still air. J. Colloid Interface Sci..

[22] Bourges-Monnier C, Shanahan MER (1995). Influence of Evaporation on Contact Angle. Langmuir.

[23] Bai Q, Huang X (2017). Effective Mass Layer of a Single Drop of Liquid Located on a Quartz Crystal Microbalance. Sensors Mater..

[24] Shin W, Nishibori M, Itoh T, Izu N, Matsubara I (2008). Monitoring of dispensed fluid with the quartz crystal microbalance (QCM) for the better control of inkjet or dispenser machine. J. Ceram. Soc. Jpn.

[25] Cumpson PJ, Seah MP (1990). The quartz crystal microbalance; radial/polar dependence of mass sensitivity both on and off the electrodes. Meas. Sci. & Technol..

[26] Bácskai J, Láng G, Inzelt G (1991). Quartz crystal microbalance response of polymer films with uneven thickness coated on electrodes. J. Electroanal. Chem..

[27] Stalder A (2010). Low-bond axisymmetric drop shape analysis for surface tension and contact angle measurements of sessile drops. Colloids and Surfaces A: Physicochem. Eng. Aspects.

[28] McKenna L, Newton MI, McHale G, Lucklum R, Schroeder J (2001). Compressional acoustic wave generation in microdroplets of water in contact with quartz crystal resonators. J. Appl. Phys..

[29] Sauerbrey G (1959). Verwendung von Schwingquarzen zur Wägung dünner Schichten und zur Mikrowägung. Zeitschrift für Physik.

[30] Kanazawa KK, Gordon JG (1985). Frequency of a quartz microbalance in contact with liquid. Anal. Chem..

[31] Keiji Kanazawa K, Gordon JG (1985). The oscillation frequency of a quartz resonator in contact with liquid. Anal. Chimica Acta.

[32] Martin SJ, Frye GC, Ricco AJ, Senturia SD (1993). Effect of Surface Roughness on the Response of Thickness-shear Mode Resonators in Liquids. Anal. Chem..

[33] Heusler KE, Grzegorzewski A, Jäckel L, Pietrucha J (1988). Measurement of Mass and Surface Stress at One Electrode of a Quartz Oscillator. Berichte der Bunsengesellschaft für physikalische Chemie.

[34] Thompson M, Kipling AL, Duncan-Hewitt WC, Rajaković LV, Čavić-Vlasak BA (1991). Thickness-shear-mode acoustic wave sensors in the liquid phase. A review. The Analyst.

[35] Rodahl M, Höök F, Kasemo B (1996). QCM operation in liquids: An explanation of measured variations in frequency and Q factor with liquid conductivity. Anal. Chem..

[36] Lin Z, Ward MD (1995). The Role of Longitudinal Waves in Quartz Crystal Microbalance Applications in Liquids. Anal. Chem..

[37] Hess C, Borgwarth K, Heinze J (2000). Integration of an electrochemical quartz crystal microbalance into a scanning electrochemical microscope for mechanistic studies of surface patterning reactions. Electrochimica Acta.

[38] Schneider TW, Martin SJ (1995). Influence of Compressional Wave Generation on Thickness-Shear Mode Resonator Response in a Fluid. Anal. Chem.

[39] Couturier G, Boisgard R, Jai C, Aimé JP (2007). Compressional wave generation in droplets of water deposited on a quartz crystal: Experimental results and numerical calculations. J. Appl. Phys..

[40] Martin BA, Hager HE (1989). Flow profile above a quartz crystal vibrating in liquid. J. Appl. Phys..

[41] Martin BA, Hager HE (1989). Velocity profile on quartz crystals oscillating in liquids. J. Appl. Phys..

[42] Comsol multiphysics v. 5.3a. www.comsol.com. comsol ab, stockholm, sweden.

[43] Erturk, A. & Inman, D. J. *Piezoelectric Energy Harvesting: Modelling and Application*, 1 edn (John Wiley & Sons, Incorporated, 2011).

[44] Holmes M J, Parker N G, Povey M J W (2011). Temperature dependence of bulk viscosity in water using acoustic spectroscopy. Journal of Physics: Conference Series.

[45] Rumble, J. R. (ed.) *CRC Handbook of Chemistry and Physics* 98 edn (CRC Press/Taylor & Francis, Boca Raton, FL, 2017).

[46] Volk A, Kähler CJ (2018). Density model for aqueous glycerol solutions. Exp. Fluids.

[47] Cheng NS (2008). Formula for the viscosity of a glycerol-water mixture. Ind. Eng. Chem. Res..

