# Supplementary material for: The Role of Wettability on the Response of a Quartz Crystal Microbalance Loaded with a Sessile Droplet
Source: Sci Rep. 2019 Nov 21;9:17289. doi: 10.1038/s41598-019-53233-y (PMC6872598; doi:10.1038/s41598-019-53233-y)
Supplement: Supplementary file 1 — Supplementary Information [file 41598_2019_53233_MOESM1_ESM.pdf]

The Role of Wettability on the Response of a Quartz Crystal Microbalance loaded with a Sessile Droplet

Brandon Murray[1] **and Shankar Narayanan**[1,*]

[1]Rensselaer Polytechnic Institute, Department of Mechanical Aerospace and Nuclear Engineering, 110 8th Street, Troy, NY 12180, USA
[*]narays5@rpi.edu

SUPPLEMENTAL MATERIAL

Meshing

Meshing of the piezoelectric and fluid domains was performed to refine areas of interest, with the most critical regions being the three-phase contact line of the droplet and the interface between the fluid and the piezoelectric domains. In Fig. S1 this area is examined, hexahedral elements are swept in a circular arc around at the contact line of the droplet with small dimensions in the radial direction (where a high rate of change is expected) and longer dimensions in the azimuthal direction. Prismatic elements are used for the solid QCM domain, and some of the bottom of the droplet while pyramidal elements join the hexahedrons to the tetrahedrons used in the upper portion of the droplet.

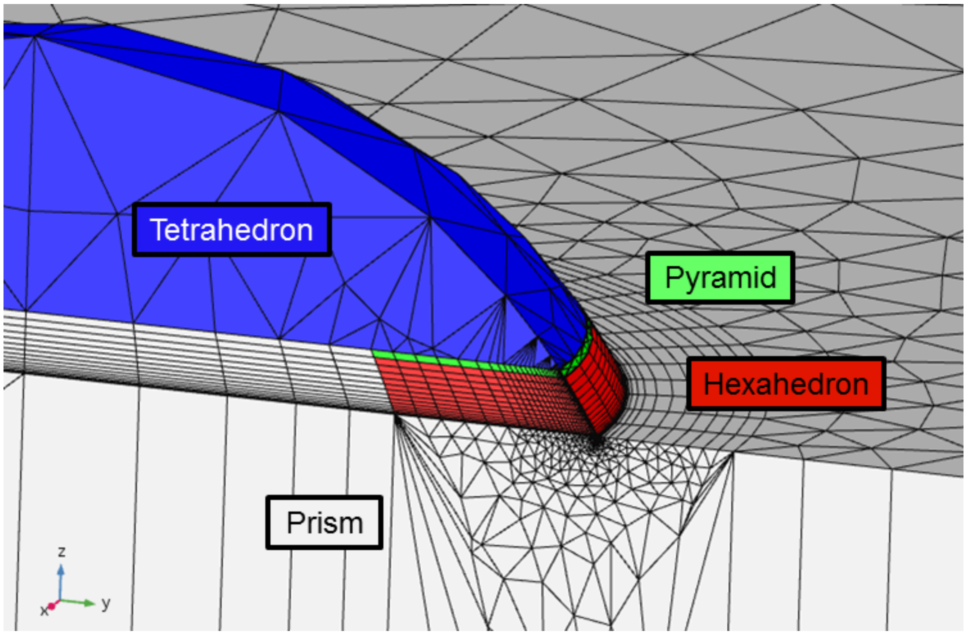

Supplemental Material Figure S1. Mesh elements near contact line in cutaway of 60°droplet on QCM.

Supplemental Material Table S1. Properties of Water for Computational Model, $T = 25°C$[1,2].

ρ	= 997	kg/m^3	μ	= 890.04	μPa·s	μ_B	= 2.4852	mPa s
c	= 1496.5	m/s	C_v	= 4.1379	kJ/kg/K	C_p	= 4.1816	kJ/kg/K
σ	= 71.97	mN/m	k	= 606.46	mW/m/K	β_T	= 4.5424×10^{-4}	1/MPa

Supplemental Material Table S2. Properties of Glycerol for Computational Model, $T = 25°C$[1]. Isothermal compressibility, β_T, was only available at 20°C, and no reliable data for bulk viscosity, μ_B, could be obtained.

ρ	= 1259.8	kg/m^3	μ	= 934	$\mu Pa \cdot s$	μ_B	= 0 (no data)	mPa s
c	= 1904	m/s	C_v	= 2.377	kJ/kg/K	C_p	= 2.377	kJ/kg/K
σ	= 62.5	mN/m	k	= 285	mW/m/K	β_T	$= 2.1 \times 10^{-5}$	1/MPa (20°C)

Supplemental Material Table S3. Physical QCM dimensions (in mm)

	Quartz Radius	Upper Electrode Radius	Lower Electrode Radius	Height
5 MHz	12.7	6.4	3.2	0.3318
10 MHz	7	2.5	2.5	0.16635

Material Properties

Quartz Strain-Charge Properties[3]:

$$s_E = \begin{bmatrix} 12.77 & -1.79 & -1.22 & -4.5 & 0 & 0 \\ -1.79 & 12.77 & -1.22 & 4.5 & 0 & 0 \\ -1.22 & -1.22 & 9.6 & 0 & 0 & 0 \\ -4.5 & 4.5 & 0 & 20.0 & 0 & 0 \\ 0 & 0 & 0 & 0 & 20.0 & -9 \\ 0 & 0 & 0 & 0 & -9 & 29.12 \end{bmatrix} \cdot 10^{-12}\ 1/Pa$$

$$d = \begin{bmatrix} -2.307 & 2.307 & 0 & 0.725 & 0 & 0 \\ 0 & 0 & 0 & 0 & -0.725 & 4.6 \\ 0 & 0 & 0 & 0 & 0 & 0 \end{bmatrix} \cdot 10^{-12}\ C/N$$

$$\varepsilon_r = \begin{bmatrix} 4.514 & 0 & 0 \\ 0 & 4.514 & 0 \\ 0 & 0 & 4.634 \end{bmatrix}$$

Animations

Combined Stress: This animation shows the normalized stress profiles ($\widetilde{T}_{zx}$, $\widetilde{T}_{zy}$, $\widetilde{T}_{zz}$) as they vary over space and time during a period of oscillation for a 10 μm radius water droplet of contact angle 30°, 60°, and 90°calculated by the coupled model of a 10 MHz QCM.

Non-dimensional Wall Stress: This animation compares the normalized stress $\widetilde{T}_{zx}$ for a 10 μm radius water droplet of contact angles 30°, 60°, and 90°along a line 45° from the x-axis calculated using the coupled model of a 10 MHz QCM.

Comparing $\widetilde{T}_{zx}$, $\widetilde{T}_{zy}$, and $\widetilde{T}_{zz}$: These animations depict $\widetilde{T}_{zx}$, $\widetilde{T}_{zy}$, and $\widetilde{T}_{zz}$ respectively along a line 45° from the x-axis for both the coupled and decoupled models. The stress calculated from both models is in agreement for the 10 μm water droplet shown at 30° and 60°contact angles on a 10 MHz QCM.

References

1. Rumble, J. R. (ed.) *CRC Handbook of Chemistry and Physics* (CRC Press/Taylor & Francis, Boca Raton, FL, 2017), 98 edn.
2. Holmes, J., Parker, G. & Povey, W. Temperature dependence of bulk viscosity in water using acoustic spectroscopy. In *Journal of Physics: Conference Series*, vol. 269, DOI: 10.1088/1742-6596/269/1/012011 (2011). 1002.3029v1.
3. Comsol multiphysics® v. 5.3a. www.comsol.com. comsol ab, stockholm, sweden.

Legends

Figure S1: Mesh elements near contact line in cutaway of 60°droplet on QCM.

Table S1: Properties of Water for Computational Model, $T = 25°C$[1,2].

Table S2: Properties of Glycerol for Computational Model, $T = 25°C$[1]. Isothermal compressibility, β_T, was only available at 20°C, and no reliable data for bulk viscosity, μ_B, could be obtained.

Table S3: Physical QCM dimensions (in mm)
