# Supplementary figures and images for: The Role of Wettability on the Response of a Quartz Crystal Microbalance Loaded with a Sessile Droplet
Source: Sci Rep. 2019 Nov 21;9:17289. doi: 10.1038/s41598-019-53233-y (PMC6872598; doi:10.1038/s41598-019-53233-y)

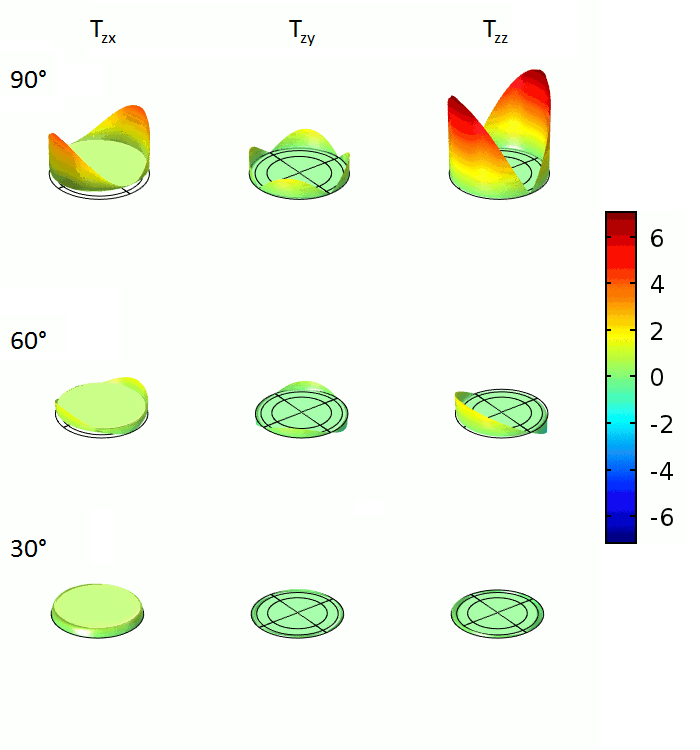

Supplement: Supplementary file 2 — Combined Stress [file 41598_2019_53233_MOESM2_ESM.gif]

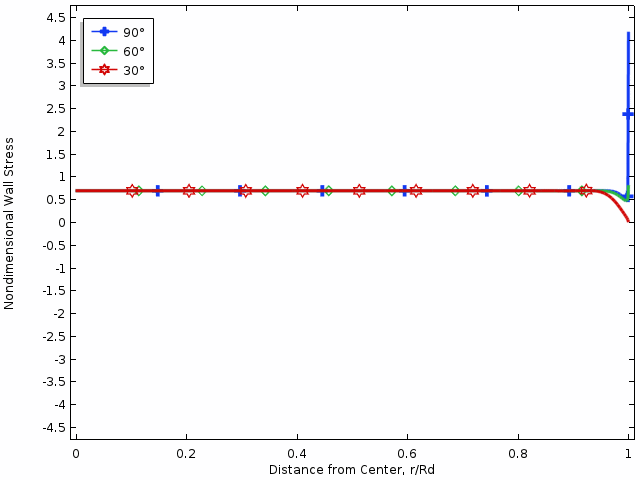

Supplement: Supplementary file 3 — Nondimensional Wall Stress [file 41598_2019_53233_MOESM3_ESM.gif]

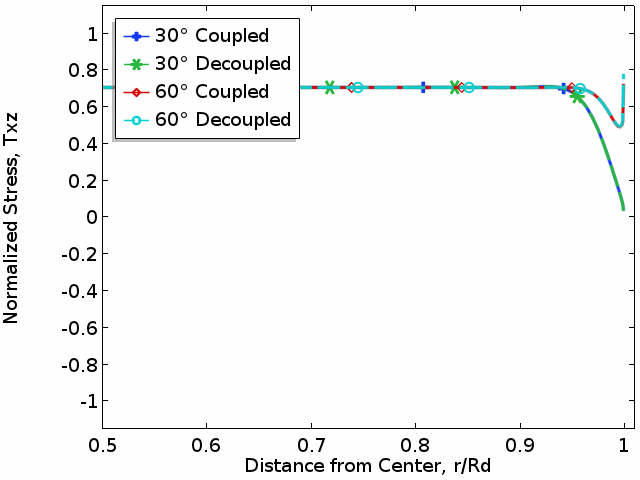

Supplement: Supplementary file 4 — Comparing Tzx for Coupled and Decoupled Analysis [file 41598_2019_53233_MOESM4_ESM.gif]

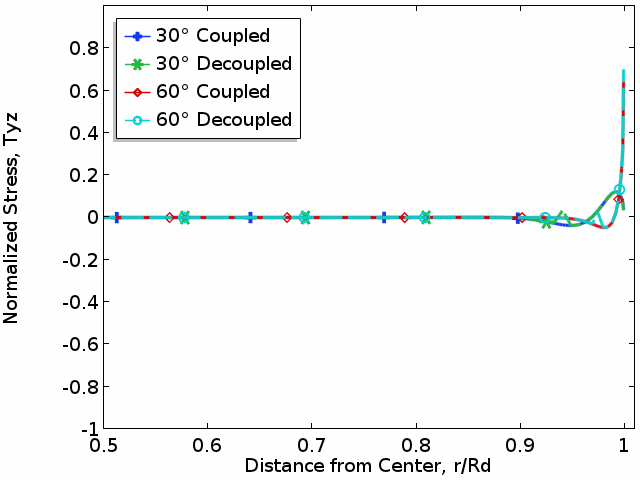

Supplement: Supplementary file 5 — Comparing Tzy for Coupled and Decoupled Analysis [file 41598_2019_53233_MOESM5_ESM.gif]

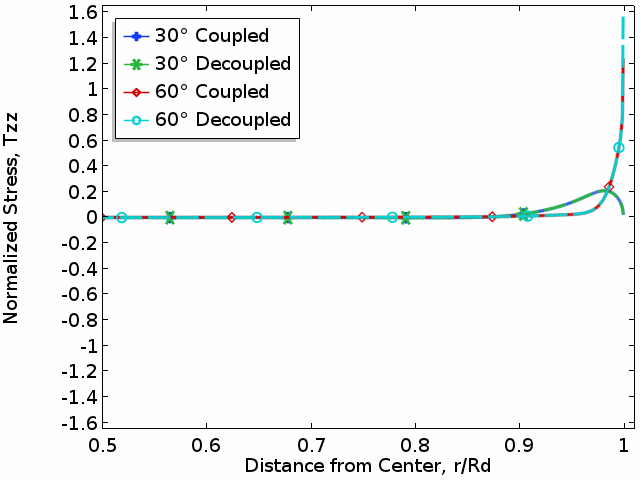

Supplement: Supplementary file 6 — Comparing Tzz for Coupled and Decoupled Analysis [file 41598_2019_53233_MOESM6_ESM.gif]
